# Puerperium could have better intensive care outcomes with acute respiratory distress syndrome caused by COVID-19

**DOI:** 10.1097/MD.0000000000031077

**Published:** 2022-10-14

**Authors:** Evrim Kucur Tulubas, Halil Çetingök

**Affiliations:** a Bakirköy Dr.Sadi Konuk Training and Research Hospital, Department of Anesthesia and Reanimation, Istanbul, Turkey; b İstanbul University, Department of Anesthesia and Reanimation, Istanbul, Turkey.

**Keywords:** COVID-19- induced ARDS, intensive care unit, postpartum women, puerperium

## Abstract

Acute respiratory distress syndrome (ARDS) with (COVID-19 often result in mortality. Treatment outcomes among puerperant, when compared with non-pregnant women (NPW) with the same syndrome. Physiological changes underwent within the gestation period have a considerable impact on the immune system, respiratory system, cardiovascular function, and coagulation. Through this research, it was aimed to compare intensive care unit (ICU) follow-up and treatment results of postpartum-period patients with those of non-pregnant ones. During the first week of ICU, 23 puerperant COVID-19 patients with ARDS and 34 non-pregnant COVID-19 patients took part in the study. Age, height, and predictive body weight (PBW) at admission to the ICU were compared with the clinical parameters of disease severity, such as FiO2 (fraction of inspired oxygen), PaO2 (arterial oxygen partial pressure), Horowitz index (PaO2/FiO2), procalcitonin (PCT), and C-reactive protein (CRP). Respiration parameters were recorded a meta-vision back server. Demographic data, FiO2, PaO2, Horowitz index, PCT, CRP and respiration parameters values were similar in both groups. The duration of non-mechanical ventilation and number of patients were seen to be significantly greater among the puerperant group than control group (*P*; .04 and .002, respectively). The duration of mechanical ventilation was similar in both groups (*P*; .07), while the mortality rate was lower in the puerperant group (*P*; .004). The postpartum women with COVID-19 induced ARDS were observed to have better ICU follow-up results and lower mortality. However, it is considered that the present results need to be supported greater number of participants.

## 1. Introduction

The new type of coronavirus (COVID-19) has been the first major pandemic of the millennium. Since February 27, 2020, it has become a global crisis resulting in hundreds of millions of cases and mortality.^[1]^

Dynamic monitoring and supportive treatment are of substantial importance for the recovery of multiple organ dysfunction. The infection is concomitant with serious respiratory problems and requires intensive care unit (ICU) treatment for the COVID-19 patients.^[2,3]^

Pregnant and puerperant (postpartum) women go through changes within their immune systems, which can make them more susceptible to respiratory viruses. It has been argued that the disease follows a different course in pregnant women due to their altered immunity and response to viral infections.^[4]^ The course and consequences of the disease remain uncertain among pregnant women infected with SARS-CoV-2.^[5]^ Compared to non-pregnant women (NPW) of reproductive age, pregnant and puerperant women with COVID-19 have been shown to be more likely to need further treatment within ICU and require respiratory support.^[6]^

COVID-19 can lead to comorbidities, especially acute respiratory distress syndrome (ARDS). ARDS is a heterogeneous syndrome with high mortality. The difference between COVID-19 related ARDS and the non-related type is assumed to be hypersensitivity reactions accompanied by micro coagulopathy with high inflammatory response. Therefore, a thorough understanding of the COVID-19 induced ARDS characteristics is essential for early diagnosis and definitive treatment.^[7]^

An altered inflammatory response to protect the fetus from maternal rejection during pregnancy may take part in alleviating the severity of that extreme reaction against self-organs. In this study, it was aimed to compare the ICU follow-up and treatment outcomes of postpartum women and NPW with similar demographic and initial clinics who suffered COVID-19 induced ARDS.

## 2. Materials and Methods

### 2.1. Study design and data collection

The researchers obtained the data of 591 COVID-19 patients hospitalized in the ICU of Istanbul Bakirköy Dr Sadi Konuk Training and Research Hospital between March 18, 2020 and November 1, 2021 from the hospital registry system. They exempted 372 male and 112 female cases over 45 ages from the study. Fifty female patients, on the other hand, were excluded because of the ICU stay shorter than 48 hours. Eventually, of the remaining 57 female patients of reproductive age, all of whom suffered ARDS, 23 postpartum COVID-19 patients and 34 non-pregnant COVID-19 patients were included in the study.

### 2.2. COVID-19 diagnosis

The diagnosis of COVID-19 was made based on the thorax computed tomography (CT) findings and nasal swab polymerase chain reaction (PCR) test results (Bio-Speedy COVID-19 RT-qPCR detection Kit ‐ Bioeksen R&D Technologies, Turkey). All patients yielded typical radiological findings consistent with COVID-19-induced ARDS, while 12 of the postpartum women and 23 of the NPW turned out to be PCR positive.

### 2.3. ARDS diagnosis

The diagnosis of ARDS was made with respect to the Berlin criteria, which include the following: presence of acute hypoxemic respiratory failure (AHRF) (PaO2/FiO2 < 300 mm Hg, PEEP > 5 cm H2O), sudden onset or worsening of respiratory symptoms within one week, bilateral opacities appeared on chest X-ray or CT not fully explained by pleural effusions, lobar collapse, lung collapse, or pulmonary nodules, and acute respiratory failure not merely explained by cardiac failure.^[8]^

### 2.4. Homogeneity of study groups

Patients were compared according to age, height, and predictive body weight (PBW) parameters as well as Horowitz index (arterial oxygen partial pressure (PaO2/FiO2)), procalcitonin (PCT), and C-reactive protein (CRP) values at admission, which are among the clinical parameters of disease severity. Some prognostic values such as D-dimer, fibrinogen, and ferritin are known to be probable to indicate physiological changes during pregnancy.^[9]^ Therefore, those parameters were kept outside the study scope. Most of the pregnant women were discharged within a week upon being admitted to the ICU. In this regard, the mean weekly values of prognostic factors, revealing the clinical course of the disease, were compared.

### 2.5. Respiratory parameters

All patients receiving orotracheal intubation were ventilated in pressure-controlled ventilation (PCV) mode through a Maquet Servo-i (Sweden) ventilator. Respiratory parameters, including end-inspiratory peak pressure (Ppeak), airway inspiratory pressure (driving pressure, DP), positive end-expiratory pressure (PEEP), mean airway pressure (Pmean), respiratory rate (RR), expiratory tidal volume (∆V), inspiratory time (Tinsp), compliance (C, calculated by ventilator: expiratory tidal volume/end-inspiratory pressure ‐ PEEP) and inspiratory-expiratory ratio (I:E ratio), were obtained through mechanical ventilators when under sedation, and PCV within the ICU. Patients’ respiratory parameters were obtained through the values on the ventilator generated at zero value of each minute and recorded on the database via the driver (Meta vision back server) customized for integration.

The non-intubated patients were alternately applied High-flow nasal cannula (Dräger Hi-Flow Star HFNC Solution, USA) and continuous positive airway pressure (CPAP). The mean flow rate was 20‐60 lt, while the FiO2 ranged from 30% to 60% among the patients who were applied HFNC.

### 2.6. Statistical methods

GraphPad Prism (v 5.01) software was used for the statistical analysis of the study findings. The variable homogeneity was assessed through the Shapiro–Wilk normality test. Since the number of COVID-19 infected pregnant patients was below 30 and the data were not homogeneous, the Mann–Whitney *U* test was performed for pair-wise comparisons of group parameters. The frequency and percentages of categorical variables were compared through Chi-square test. Median (Inter Quantile Range 25‐75) values were used for statistical notation. Statistical values with a *P*-value of .05 were considered significant.

### 2.7. Ethical approval

The study was approved by Clinical Research Ethics Committee, Health Sciences University Bakirköy Dr Sadi Konuk Training and Research Hospital, with the decision numbered 2021/473. During the ICU admission, patient relatives were told in detail that the patient data could be used for scientific purposes retrospectively, and patient consent forms were obtained.

## 3. Results

Patients suffering acute respiratory distress within a week were seen to have bilateral lung opacities on CT, though they had no cardiac problems. The Horowitz index was below 300 and the peep applied was above 5 cm H2O.

Height, age, PBW, FiO2, PaO2, Horowitz index, PCT, and CRP values on ICU admission were found to be similar in both groups (namely puerperant and non-pregnant COVID-19 patients). Of those parameters, the median, inter-quartile range, and *P* values are as seen in Table 1.

**Table 1:** Demographic data and intensive care unit (ICU) admission parameters.

Parameter	Puerperant group	Non-pregnant group	*P* value
Age	33 (30-34)	32,5 (26,75-38)	.921
Height	165 (163-166)	164 (160-165)	.224
PBW	57 (55-58)	56 (52-57)	.236
FiO2	46 (40-55)	54 (40-66)	.952
PaO2	87 (72-117)	94 (70-111)	.84
Horowitz index	181 (119-280)	185 (111-272)	.555
PCT	0,5 (0.2-0.8)	0.7 (0.2-4.3)	.21
CRP	90 (45-129)	80 (15-166)	.5

CRP = C-reactive protein, FiO2 = fraction of inspired oxygen, PaO2 = arterial oxygen partial pressure, PBW = predictive body weight, PCT = procalcitonin.

During the first week of ICU, the mean median values of mechanical ventilator parameters were found to be respectively FiO2: 44.3–46.4, Ppeak: 25.6–25.5, and PEEP: 8–8.2, showing similarity among the puerperant and non-pregnant patients. Compliance was relatively higher among the postpartum patients, with no statistical difference, whereas, accordingly, tidal volumes were significantly higher. Among the mean blood gas values per week, the mean pH and arterial oxygen partial pressure (PaO2) were determined to be similar while arterial partial carbon dioxide pressure (PaCO2) and bicarbonate (HCO_3_) were significantly lower compared to the non-pregnant COVID-19 patients. Among biochemical prognostic factors, PCT and CRP values were similar in both groups (Table 2).

**Table 2:** One-week mean values of prognostic findings.

Parameter	Puerperant group	Non-Pregnant group	*P* value
FiO2	44.3 (41.2-50)	46.4 (39.1-61.8)	.654
Ppeak	25.6 (23-27.5)	25.5 (21.5-28.3)	.785
PEEP	8 (7.6-9.4)	8.2 (6.7-9.6)	.975
Compliance	36 (25-42)	25 (14-34)	.06
DP	15.2 (11.3-18.3)	15.7 (13-18.4)	.584
Tv	523 (459-561)	409 (256-456)	<.001
PH	7.42 (7.41-7.45)	7.42 (7.40-7.44)	.4
PaO2	86.4 (78.5-110.2)	93.8 (77.6-111.1)	.839
PCO2	35 (32-40)	47 (41-49)	<.001
HCO_3_	24 (24-26)	29 (26-31)	<.001
SPO2	96 (93-97)	96 (93-98)	.877
PCT	0.4 (0.1-0.6)	1.1 (0.1-7.6)	.063
CRP	71.9 (38.6-139.4)	73.9 (30.3-160.6)	.915

FiO2 = fraction of inspired oxygen, Ppeak = end-inspiratory peak pressure, PEEP = positive end-expiratory pressure, DP = driving pressure, TV = tidal volume, PaO2 = arterial oxygen partial pressure, PaCO_2_ = arterial partial carbondioxide pressure, HCO_3_ = bicarbonate, SpO_2_ = oxygen saturation, PCT = procalcitonin, CRP = C-reactive protein.

When the duration of weaning from mechanical ventilation, which means discontinue the mechanical support, the number of patients who underwent HFNC + CPAP were compared, the duration length was statistically found to be significantly higher in the puerperant group (*P*-value of each group was .04 and .002 respectively).

As seen in Table 3, while the duration of mechanical ventilation in each group was similar (*P*; .07), the mortality in the puerperant group was statistically noted to be significantly lower when compared to the non-pregnant group (*P*; .004).

**Table 3:** Mortality, durations of ICU stay and mechanical ventilation (MV).

Parameter	Puerperant group	Non-pregnant group	*P* value
Duration of ICU stay (day)	4.8 (2.9-9.6)	8.5 (2.9-19.2)	.1
Duration of MV (day)	4.8 (1.3-9.4)	7 (4.5-19.8)	.07
Duration of weaning from MV (day)	3.2 (1-4.3)	1.2 (0.3-2.9)	.04
HFNC + CPAP therapy (%)	15 (%65.2)	8 (%23.5)	.002
ICU mortality (%)	2 (%8.8)	15 (%40.1)	.004

CPAP = continuous positive airway pressure, HFNC = high-flow nasal cannula, ICU = intensive care unit, MV = mechanical ventilation.

## 4. Discussion

Having similar demographics and clinical characteristics at ICU admission, the puerperant women, who developed COVID-19 induced ARDS, displayed better ICU follow-up and treatment outcomes when compared to NPW of reproductive age.

Blitz et al^[10]^ compared COVID-19-induced ICU admissions of non-pregnant (n = 332; age, 15‐49) and pregnant women (n = 82), reporting that pregnant women were not at elevated risk for ICU admission. In that study, 15.1% of NPW and 9.8% of pregnant patients were admitted to the ICU (*P* = .22). Similarly, Savasi et al^[11]^ reported that only 6 out of totally 77 COVID-19 infected pregnant women were admitted to the ICUs of 12 hospitals in Italy, and eventually, all patients fully recovered.

On the contrary, two studies^[12,13]^ reported that pregnant women with COVID-19 had a higher risk of severe illness compared to NPW. A Swedish study indicated that pregnant women were five times more likely to be admitted to the ICU and four times more likely to receive MV than NPW.^[13]^

Evaluating the findings of current research, in which postpartum women and NPW of childbearing age infected with COVID-19 were compared, the mortality was determined to be 8.8% among the postpartum women with moderate ARDS, being attributed to SARS-CoV-2 with reference to the Berlin criteria, while it was 40.1% among the NPW. Additionally, the length of ICU stay was shorter among the puerperant women patients. Though not statistically significant, the duration of MV was shorter among those postpartum women. ProBNP, Ferritin, and LDH values monitored during the ICU stay were found to be significantly higher in the non-pregnant group as expected. A study with equivalent results to those of this research was published in Brazil in 2002, where pregnant and puerperant women with severe COVID-19 induced ARDS exhibited a lower mortality rate compared to NPW, despite associated comorbidities. COVID-19-associated ARDS mortality was reported as 7.8% (377/4853) for the obstetric group and 13.9% (5946/42 915) for the non-obstetric group.^[14]^ In the present research, 2 deaths occurred among the postpartum women; however, the number of cases included was higher in the above-mentioned paper. A cohort study in the USA examined critically ill pregnant patients, reporting that ARDS occurred at a rate of 70% (14 of 20), reintubation was needed at a rate of 20% (4 out of 20), and no case of cardiomyopathy or maternal death.^[15]^

Various phenotypes of COVID-19 induced ARDS are in question within the clinical course of the disease, where periods of increased vascular permeability and hyper-inflammation, resulting in hypoxic pulmonary coagulopathy and micro-thrombosis, are observed.^[16]^ It is assessed that the better response of pregnant women with ARDS to treatment is due to the fact that the immunosuppression, being caused by pregnancy, inhibits the COVID-induced hyper inflammation process. The reason may be the maternal immunosuppressive factors persisting before, during, or shortly after delivery. A considerable number of researchers have reported that immunity is suppressed during pregnancy in several ways^[17,18]^ which can be detected through immunophenotypic and functional tests.^[19,20]^ Such immune changes are considered to involve a complex interaction of pregnancy-related hormonal changes and the immune system.^[21]^

## 5. Limitations

That there was a small number of patients being evaluated through retrospective analysis, not all patients were followed up with invasive mechanical ventilator, and laboratory parameters indicating immunosuppression were absent have been considered as the main limitations of the present research. Having examined a specific patient group, the puerperant ICU patients with COVID-19 induced ARDS, the present research is the first study ever made in the field, and this can be assumed as one of its strengths. Furthermore, the Metavision back server-assisted data collection, being fulfilled based on objective, continuous, reliable measurements, has been another strength for this study.

## 6. Conclusion

According to the research results, postpartum women with COVID-19 induced ARDS exhibited better ICU follow-up outcomes and lower mortality compared to the control group. It is believed that further studies need to be conducted to identify the mechanisms to support such findings, as well as the consequences resulting from the immune response changes occurring in the pregnancy and the postpartum period.

## Author contributions

**Conceptualization:** Evrim Kucur Tulubas.

**Data curation:** Evrim Kucur Tulubas.

**Formal analysis:** Evrim Kucur Tulubas.

**Funding acquisition:** Evrim Kucur Tulubas.

**Investigation:** Evrim Kucur Tulubas.

**Methodology:** Evrim Kucur Tulubas, Halil Cetingök.

**Software:** Evrim Kucur Tulubas.

**Supervision:** Halil Cetingök

**Project administration:** Evrim Kucur Tulubas.

**Resources:** Evrim Kucur Tulubas.

**Validation:** Halil Cetingök.

**Writing – original draft:** Evrim Kucur Tulubas.

**Writing – review & editing:** Evrim Kucur Tulubas.
